# Rhabdomyosarcoma of the Cervix in a Post-Menopausal Woman—An Unparalleled Phenomenon

**DOI:** 10.3390/ijerph18157851

**Published:** 2021-07-24

**Authors:** Jakub Pawlik, Weronika Pawlik, Dorota Branecka-Woźniak, Katarzyna Kotrych, Aneta Cymbaluk-Płoska

**Affiliations:** 1 Department of Gynecological Surgery and Oncology of Adults and Adolescents, Pomeranian Medical University, 70-111 Szczecin, Poland; weronikapawlik7@gmail.com (W.P.); aneta.cymbaluk@gmail.com (A.C.-P.); 2 Department of Gynecology and Reproductive Health, Pomeranian Medical University, 71-210 Szczecin, Poland; dobrawo@gmail.com; 3 Department of General and Dental Radiology, Pomeranian Medical University, 70-111 Szczecin, Poland; kotrych1@gmail.com

**Keywords:** rhabdomyosarcoma, sarcoma, cervix, uterus, oncology, gynecology, neoplasm, post-menopausal

## Abstract

Rhabdomyosarcoma of the cervix is a soft tissue sarcoma that usually occurs in young women. It is very rare in adulthood. We discuss symptoms, the process of diagnosis of rhabdomyosarcoma embryonale of the cervix in a 61-year-old women and differences in treatment dependent on patient’s age. A 61-year-old woman with symptoms such as palpable mass in the external cervical opening and post-menopausal hemorrhaging was admitted to the oncology ward where excision of the polyp was performed. Embryonal rhabdomyosarcoma (ERMS) was diagnosed by histopathological examination of obtained tissues. The diagnosis was complemented by chest computed tomography and pelvis magnetic resonance imaging to exclude metastases. A Wertheim–Meigs operation and excision of the ovaries, the fallopian tubes and the surrounding tissue was performed in the course of treatment. In the patient’s follow-up of 25 months to date, there have been no signs of recurrence or symptoms connected to ERMS. Based on the therapeutic outcome, the decision to limit the treatment to a surgical resection was adequate for a post-menopausal patient. Because of the rarity of ERMS in the post-menopausal age, we think that the patient should be carefully followed up to further examine this issue and develop diagnostic and treatment guidelines.

## 1. Introduction

Rhabdomyosarcoma (RMS) is the most common soft tissue sarcoma in children and adolescents [1]. It mostly occurs in patients up to 4 years old, with approximately 4 cases per 1 million children, with a lower rate in adolescents. Soft tissue sarcomas are generally unusual and unsuspected in the adult population, especially in the more mature age [2]. The most common types of soft tissue sarcoma in post-menopausal patients are: leyomiosarcoma (mostly seen in the abdomen and the uterus), fibrohistiocytic sarcoma (mostly seen in upper and lower extremities) and liposarcoma (mostly seen in the lower extremities), accounting for 19–26%, 20–25% and 12–17% of all sarcomas in these age groups, respectively [3,4]. In adults, sarcomas account only for 8% of all uterine neoplasms. They usually yield a more aggressive clinical course and lower survival rate compared to other types of uterine malignancies, such as endometrial carcinoma [5].

Rhabdomyosarcoma develops from embryonic mesenchymal cells which also differentiate into striated muscle cells [6]. It typically develops in the head and neck (approximately 25%) and the genitourinary tract area (approximately 31%). The World Health Organisation divides RMH into four types, embryonal, alveolar, pleomorphic and spindle cell/sclerosing, with embryonal being the most common type [7]. The pathophysiology of rhabdomyosarcomas still remains unclear; recent studies have shown DICER1 somatic or germ-line mutations to occur in a majority of genitourinary tract ERMS [8].

Cervical ERMS is extremely rare and there are only a few cases published in the English literature describing the management and treatment of this disease in adult women [9,10,11,12]. We report a case of a 61-year-old women with rhabdomyosarcoma embryonale of the cervix initially presenting itself as a cervical polyp. The aim of our paper is to increase awareness of such a rare neoplasm and explain the process of diagnosis. Most of all, we see the need to discuss the available treatment options and assess how to determine the best approach while dealing with various types of patients.

## 2. Case Report 

A 61-year-old woman, gravida 3 para 3, last menstruation at 54 years of age, reported to her gynecologist complaining about irregular post-menopausal hemorrhaging and a mass emerging from the external cervical opening she palpated. She presented no other symptoms or complaints, including no pain whatsoever. Her gynecologist referred her to the Obstetrics and Gynecology Clinics emergency room. On admittance, physical examination revealed no irregularities apart from a solid palpable mass in the cervix. A pre-operative imaging was performed, in this case an ultrasound examination, using a transvaginal probe, that confirmed the presence of a 5.5 cm polyp that expanded the cervix. Moreover, the patient suffered from stage 2 arterial hypertension and class 3 obesity (BMI = 40), and had undergone a single surgery in the past, an open appendectomy. Her mother suffered from a neoplasm (most likely pancreatic cancer); there were no other diseases in the family history.

**Figure 1:**
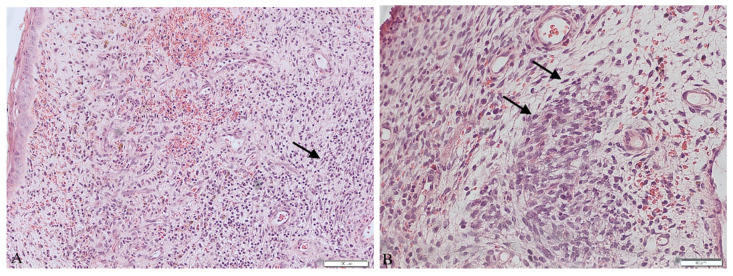
Histopathological images of the excised polyp (**A**) in 10× magnification. (**B**) in 20× magnification. Arrows show spindle and round pleomorphic cells with hyperchromatic nuclei and scant cytoplasm.

The patient underwent a surgical procedure of excising the polyp at its peduncle using a LEEP loop electrode. It could not be removed in one piece—the polyp fragmented into several pieces during extraction. Then, a curettage of the cervix and an unsuccessful attempt of a uterine cavity curettage was performed. Obtained tissues were then sent to the pathology department for a histopathological examination. The findings were ready in six weeks after the procedure and revealed the presence of rhabdomyosarcoma embryonale (type botryoides) in the excised polyp. Due to the fact that the polyp was fragmented during extraction, a precise margin status or the depth of invasion could not be properly determined. Histopathological images are presented in Figure 1.

The immunohistochemistry results are shown in Table 1. They correlate with results typically seen in ERMS.

The patient was then referred to our gynecological ward that specializes in oncology. In our outpatient clinic, a chest CT and pelvis MRI of the pelvis and the abdomen were performed and the presence of metastasis was excluded. Her treatment was then thoroughly discussed by an oncological council and further therapy was planned.

**Figure 2:**
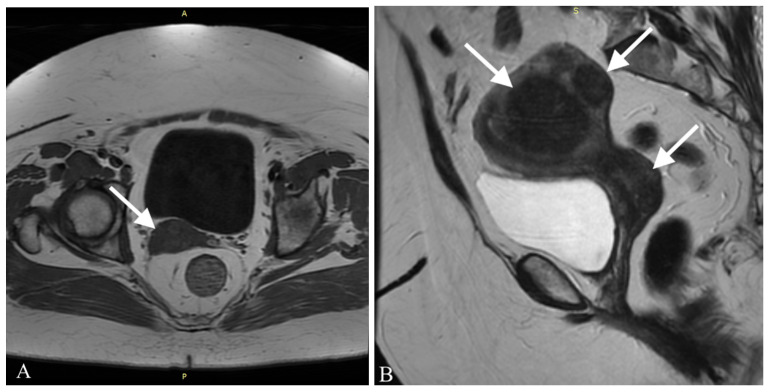
A preoperative MRI of the pelvis in (**A**) axial and (**B**) sagittal projection. Both show the presence of myomas in the uterus. Arrows show myomas in the uterus.

Three months after the initial hospitalization, the patient was admitted to the onco-logical gynecological ward. On admission she presented no irregularities in gynecological examination. A transvaginal ultrasonography of the uterus and pelvis was performed and a myoma, ca. 5 cm in diameter was found in the uterus wall. No other lesions were identified. A preoperative MRI of the abdomen and pelvis allowed identification of another 4 myomas in the uterus, with sizes from 7 to 54 mm, as shown in Figure 2.

Additionally, in the posterior wall of the cervix an irregular area (14 mm × 25 mm × 9 mm) was found, which was probably a malignant lesion. A single lymphatic node in the right inguinal area, with a diameter of 10 mm, was also identified. Remembering her symptoms and a history of a neoplasm in her family, the patient demonstrated a high level of anxiety towards her neoplasm. She expressed a wish to implement a radical treatment to ensure a disease-free result and this was also taken under consideration while choosing the therapeutic path.

We performed a Wertheim–Meigs operation—an extended uterus excision with bilateral pelvic lymphatic nodes removal. Additionally, as preserving fertility in this patient was not a priority, we excised the ovaries, the fallopian tubes and the surrounding tissue to ensure radicality of the procedure. All of the removed tissues were then forwarded to the pathology department. No adjuvant chemotherapy or radiotherapy was administered. The patient was discharged from the hospital in good general condition. 

The histopathological results showed no signs of the rhabdomyosarcoma in the excised tissues; this may indicate that all malignant lesions were placed in the polyp and were successfully removed during the initial procedure. Moreover, none of the excised lymphatic nodes showed traces of neoplasm cells.

**Figure 3:**
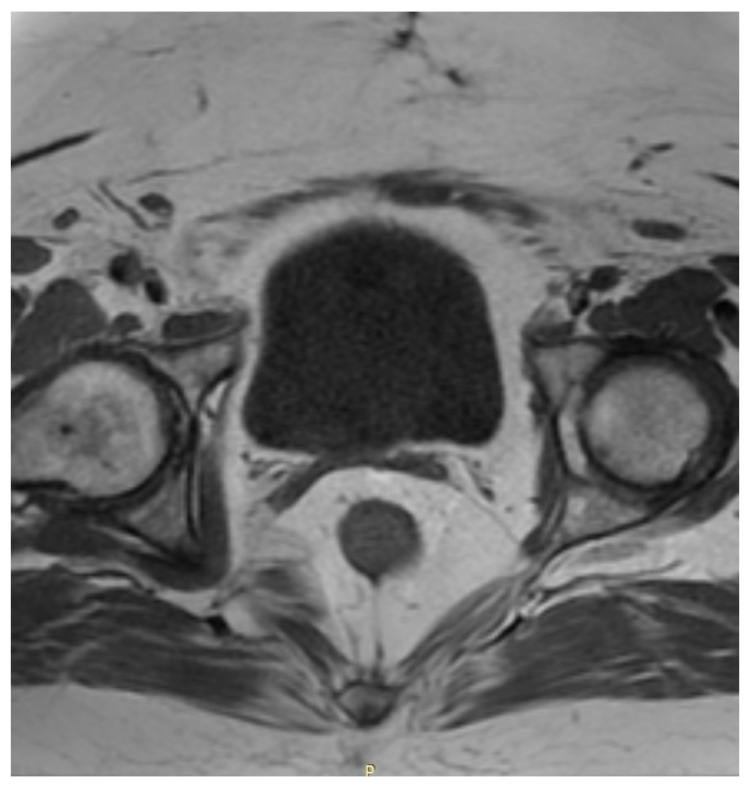
A postoperative MRI of the pelvis in axial projection, performed 18 months after the Wertheim–Meigs operation. A vaginal stump is visible. No signs of recurrence are present.

After the surgery, the patient was regularly monitored in the outpatient clinic in the first year once every 3 months and afterwards once every 6 months. Three months after the surgery, a cytological examination was performed and no irregularities were found. The patient showed no symptoms and reported no complaints. One year after the surgery a CT scan of the chest and MRI of the pelvis and abdomen were performed—with no signs of neoplasm whatsoever. A postoperative MRI of the pelvis is shown in Figure 3.

The patient still remains under our care, with biannual control visits, the same as every patient treated for a gynecological malignancy in our clinic. At her last control visit, two years after the initial surgery, she presented no symptoms and no complaints whatsoever. An ultrasound was performed in which the postoperative organ site was filled with intestinal loops; no free fluid or pathological lesions were observed. The most recent cytological examination did not show any signs of neoplastic cells. Up to this day, no signs of recurrence or symptoms connected with the ERMS were identified.

The patient’s mental and psychological status, as well as her life satisfaction after recovering from the disease was evaluated, using Beck’s Depression Inventory II, Satisfaction with Life Scale, General Self-Efficacy Scale and Coping Inventory for Stressful Situations Scale. Detailed results are presented in Table 2.

The patient shows no signs of depression or dissatisfaction regarding her current life. When encountering a problem, she presents a task-oriented coping mechanism—first she analyzes a given situation, and then deals with it, following a plan she made. Moreover, she is sure that if a problematic situation would appear, she would be capable of managing it. All this shows that our patient, despite expressing a high level of anxiety during the diagnostic and therapeutic process, is now in great mental shape and is satisfied with her life.

## 3. Discussion

We present a case of a 61-year-old woman who reported symptoms of postmenopausal bleeding and a palpable mass present in the cervix. The polyp was excised and examined, revealing the presence of an extremely rare neoplasm in this age group, i.e., embryonal rhabdomyosarcoma. After this diagnosis, the patient was referred to our department specializing in gynecological malignancies, where a radical, extended Wertheim–Meigs surgery was performed. This treatment ensured good therapeutic effect and the patient remains disease free. Given the results of this case, we demonstrate a unique clinical and pathological aspect of this neoplasm compared to its usual form of presentation. We believe that it can be very useful to medical professionals who encounter a similar, uncommon case, as this knowledge will support them in making proper diagnostic and therapeutic decisions.

Embryonal rhabdomyosarcoma of the genitourinary tract, though being a fairly common neoplasm in children and adolescents, is not a particularly frequent problem in adults. The sarcoma in adult females constitutes 0.4–1% of all cervical malignancies and is extremely rare in patients in post-menopausal age [13]. To the best of our knowledge, we present the first case of post-menopausal cervix RMS from Poland. 

The low prevalence is why there is limited knowledge about the clinical course, treatment and prognosis for ERMS in adult patients; developing a standardized treatment protocol for ERMS is difficult and is based mostly on pediatric cases and guidelines. 

The treatment administered to our patient was limited only to a surgical operation. This decision was based on the clean margins of the excised tissues, no regional or distal metastasis and embryonal histological type of sarcoma, all of which placed our patient in the low-risk category.

Post-menopausal hemorrhaging is, on the other hand, a common symptom, affecting almost 1 in 10 women worldwide. Out of all these cases, 10% are caused by neoplasms [14]. As other studies show, vaginal bleeding is also the most common symptom in RMS of the genitourinary tract. Other symptoms include abdominal and lower pelvic pain or frequent urination [9]. When dealing with patients complaining about abnormal vaginal bleeding, a careful approach is advised. Performing a histopathological examination of the tissues collected from uterine curettage is necessary, because it can allow us to diagnose even such uncommon problems as ERMS. Histopathological evaluation of the excised material is often a complicated task when dealing with sarcomas. The reason for that is the fact that sarcomas present a very diverse image. Embryonal rhabdomyosarcoma usually presents a few traits: agglomerations of spindle and round pleomorphic cells with hyperchromatic nuclei, rhabdomyoblasts and myxoid stroma [9,10,11]. Performing an immunohistochemical profile of the tissue is nowadays a must in diagnosing sarcomas. In the case of rhabdomyosarcoma, staining for myoglobin, desmin and muscle-specific actin is observed. The most sensitive and specific RMS markers are myogenin and Myo-D1, proteins characteristic for early differentiation of muscle cells, and they often prove to be the key traits in making the final diagnosis [15,16]. In our case, the immunohistochemistry showed positive CD10, myogenin and desmin and negative SMA, ER, PR and S100 markers. These results, combined with microscopic description, allowed us to perform differential diagnosis and rule out more common out neoplasms occurring more frequently, such as the leiomyosarcoma or adeno-sarcoma, as well as identify a specific subtype of RMS—rhabdomyosarcoma embryonale botryoides in this case. 

The pathogenesis of this tumor in post-menopausal women still remains unclear. In most cases it appears sporadically, but it can also be the result of a family predisposition in such genes as TP53, NF1 or HRAS. There have been studies that suggest that the DICER1 mutation which causes dysregulation of miRNA and activates oncogenes may play a part in this process [8,17,18]. A hypothesis has been formed, stating that the DICER-1 mutation is present in ERMS developing in tissues derived from Mulerrian ducts, i.e., the uterus, cervix, upper part of the vagina and the fallopian tubes [19]. Moreover, a study of 17 cases has proved that DICER-1 mutation occurs more often in older patients, while in younger ones, ERMS usually develops with no connection to that mutation. The median age of patients with DICER-1 mutation was 36 years of age vs. 5 years of age without mutation. The presence of DICER-1 mutation is also a positive prognostic factor, as it might indicate a better clinical course than mutation-free cases [8].

The prognosis of patients with rhabdomyosarcoma may vary depending on the prognostic factors. Patients can be divided into those at low and high risk of developing poor outcomes of treatment. Among the favorable factors highlighted in recent studies are: embryonal type of tumor, a superficial infiltration (<50% in MRI or <10 mm in conization material), negative histological margins of dissected tumor or no regional or distant metastases found on preoperative imaging [11]. It is extremely important to qualify patients to a specific risk group for patients of childbearing age. According to expert consensus from the Childen’s Oncology Group for treating RMS of the cervix, uteri small tumors preferably should be treated with local fertility-sparing treatment based on only partial surgical excision of the lesion, with an optional adjuvant chemotherapy [20]. This approach is based on the fact that most tumors have a good response to chemotherapy, and preserving the organ function is crucial in this group [21].

Adjuvant chemotherapy is used because of the risk of hematogenous spread of tumor cells, even in the case of early-stage sarcoma. As stated by the Intergroup Rhabdomyosarcoma Study Group (ISRG), intensive primary chemotherapy that includes vincristine, actinomycin-D and cyclophos-phamide (VAC) had better 5-year survival rates (84% for chemotherapy vs. 82% for surgery/radiation) than aggressive surgery followed by radiation [22]. This approach allows us to maintain fertility while reducing long-term morbidity from radiation therapy. The administration of chemotherapy is followed by a local surgery—a cone biopsy or polypectomy is performed. This approach has several purposes: to gather histopathological results, confirm the diagnosis, reduce tumor load and the symptoms caused by it. Radiation is mostly used for therapy in more advanced stages, recurrent disease or for adult patients who may not tolerate chemotherapy easily. 

Most of the ERMS (or in fact all RMS) of the genitourinary tract in adults is diagnosed at a late stage, with widespread disease and metastasis to the regional and distal lymphatic nodes and regions [10]. In such cases, an aggressive, multimodal treatment, combining radiotherapy and chemotherapy with a total hysterectomy with regional lymphatic nodes resection is administered [23]. Considering chemotherapy, the best results are produced when vincristine, doxorubicin and ifosfamide are combined [12,24]. However, these results are not specific to genitourinary tract ERMS. Moreover, due to low prevalence of adult sarcomas, let alone rhabdomyosarcomas, there are no standardized treatment plans for adult patients [25].

Even if patients remain in good general condition after the treatment and raise no complaints about their health, they still have to remain in our thorough follow-up. They must be instructed to attend cyclic, biannual appointments, which should include a full physical examination (with an emphasis on a gynecological examination and cytological evaluation) and ultrasonographic check. Moreover, radiological imaging, e.g., computed tomography of the chest and magnetic resonance imaging of the abdomen and pelvis should be performed to ensure there are no suspicious lesions which might indicate a recurrence. The role of the follow-up is crucial in patient wellbeing after treatment, and we must ensure that they stay free of the disease. In the case of a recurrence, we aim to find it as soon as possible, to ensure a rapid implementation of adequate treatment. The follow-up should be continued for the whole life of a patient; even though sarcomas usually recur quickly, a later recurrence could also happen [26,27]. As a postmenopausal ERMS is an unusual case, we should be also ready for its unusual clinical course.

## 4. Conclusions

Our patient has already been in follow-up for over 25 months after the initial tumor removal. So far, there are no signs of recurrence and the patient remains in good health. This could be the evidence that the treatment implemented in this case was accurate. However, as this is a very rare and unusual case of ERMS the patient still needs to be carefully monitored. Long-term follow-up of adult patients with cervical rhabdomyosarcoma is important not only to ensure their wellbeing, but also to gather more information about the pathogenesis, clinical course, prognosis and types of treatment to allow developing accurate protocols and guidelines of ERMS management. 

## Figures and Tables

**Table 1:** Immunohistochemistry findings in histopathological examination of the excised tissues.

IHC Marker	Result
Myogenin	positive
Desmin	positive
CD10	positive (focal)
Ki67	positive (70–80%)
PR	negative
ER	negative
SMA	negative
S100	negative

Abbreviations: IHC: Immunohistochemistry; CD10: neprilysin; PR: progesterone receptors; ER: estrogen receptors; SMA: smooth muscle actin.

**Table 2:** Results of mental and psychological evaluation.

Questionnaire	Score	Interpretation
Beck’s Depression Inventory II	0/63	Minimal range of depression symptoms
Satisfaction with Life Scale	31/35	Very high level of life satisfaction
General Self-Efficacy Scale	33/40	High level of self-efficacy while dealing with problems
Coping Inventory for Stressful Situations	TOC 69 EOC 46 AOC 56	Task-oriented coping with encountered problems

Abbreviations: TOC: Task-oriented Coping; EOC: Emotions-oriented Coping; AOC: Avoidance-oriented Coping.

## Data Availability

Not applicable.
